# Automated Uniform Spheroid Generation Platform for High Throughput Drug Screening Process

**DOI:** 10.3390/bios14080392

**Published:** 2024-08-15

**Authors:** Kelvin C. C. Pong, Yuen Sze Lai, Roy Chi Hang Wong, Alan Chun Kit Lee, Sam C. T. Chow, Jonathan C. W. Lam, Ho Pui Ho, Clarence T. T. Wong

**Affiliations:** 1 Department of Biomedical Engineering, The Chinese University of Hong Kong, New Territories, Hong Kong, China; kelvin.pong@link.cuhk.edu.hk; 2 BioArchitec Group Limited, Hong Kong, China; 3 State Key Laboratory of Chemical Biology and Drug Discovery, Department of Applied Biology and Chemical Technology, The Hong Kong Polytechnic University, Kowloon, Hong Kong, China

**Keywords:** 3D spheroid, drug screening, automation, microfluidic

## Abstract

Three-dimensional (3D) spheroid models are crucial for cancer research, offering more accurate insights into tumour biology and drug responses than traditional 2D cell cultures. However, inconsistent and low-throughput spheroid production has hindered their application in drug screening. Here, we present an automated high-throughput platform for a spheroid selection, fabrication, and sorting system (SFSS) to produce uniform gelatine-encapsulated spheroids (GESs) with high efficiency. SFSS integrates advanced imaging, analysis, photo-triggered fabrication, and microfluidic sorting to precisely control spheroid size, shape, and viability. Our data demonstrate that our SFSS can produce over 50 GESs with consistent size and circularity in 30 min with over 97% sorting accuracy while maintaining cell viability and structural integrity. We demonstrated that the GESs can be used for drug screening and potentially for various assays. Thus, the SFSS could significantly enhance the efficiency of generating uniform spheroids, facilitating their application in drug development to investigate complex biological systems and drug responses in a more physiologically relevant context.

## 1. Introduction

Three-dimensional (3D) cancer cell spheroids have emerged as a more advanced cell culture system compared to traditional monolayer cell cultures [1,2]. These spheroids can replicate the complex 3D structure of tumour tissues, offering a more realistic model for cancer research [3]. Three-dimensional (3D) spheroids can mimic the cellular environment within tumours, allowing for cell–cell interactions and the gradient diffusion of nutrients and drugs. One of the key advantages of 3D spheroids is their ability to provide insights into drug resistance [4,5]. Often, 3D cultures show higher resistance to drugs compared to monolayer cell cultures, as they better simulate the diffusion barriers and microenvironmental influences found in tumours. Moreover, 3D spheroids enable a deeper understanding of a drug’s mechanism of action, toxicity, penetration, and metabolism in a tissue-like context, which is not possible with monolayer cell cultures [6,7]. As a result, they have become crucial tools for studying tumour biology and drug discovery and are potentially used to identify candidates for personalized therapy [8].

Despite the numerous advantages of 3D spheroids in cancer research, several technical limitations hinder their widespread use. Each of the traditional methods for producing 3D spheroids has its own advantages and limitations [9,10,11]. For example, the hanging drop method involves suspending small droplets of cell suspension from the lid of a Petri dish, allowing cells to aggregate and form spheroids at the bottom of the droplet. While this method is simple and cost-effective, it is labour-intensive and has limited scalability and consistency [12,13]. The liquid overlay technique uses non-adherent surfaces, such as agarose-coated plates, to prevent cell attachment and promote spheroid formation. Although this method is straightforward, it often results in heterogeneous spheroid sizes and shapes [14]. Another approach is the use of rotating bioreactors, which provide a dynamic culture environment for spheroid formation. However, this method requires specialized equipment and may not be suitable for high-throughput applications [15]. Furthermore, the inherent mechanical weakness of spheroids also adds complexity, as they are prone to damage during physical handling, particularly in automated settings.

Recent advancements in 3D cell line spheroid and organoid generation have focused on addressing these limitations and improving uniformity and reproducibility [16,17,18,19,20,21,22,23,24]. For example, Behroodi et al. reported a microfluidic device that combines 3D printing and computer numerical control (CNC) micro-milling to create microwells of varied depths for tumour spheroid production [25]. Jung et al. designed a microfluidic device for one-stop organoid growing and drug testing with higher uniformity and reproducibility than the traditional Matrigel method [26]. Grexa et al. introduced the SpheroidPicker, an automated 3D cell culture manipulator, although its slower speed limits high-throughput screening [27]. Ochoa and co-workers developed SpheroidJ, an open-source tool for adaptable spheroid segmentation, improving analysis reliability [28]. Piccinini, in 2015, reported the AnaSP software (source code and a standalone executive version 2015 can be found at: https://sourceforge.net/projects/anasp/, accessed on 11 August 2024) for automatic image analysis of spheroids [29]. Deep learning has become a recent trend to assist spheroid research [30]. Despite the advancements in 3D spheroid generation, a comprehensive system that can efficiently produce uniform spheroids in a high-throughput manner while simultaneously facilitating their sorting and application in drug screening assays remains lacking.

**Scheme 1:**
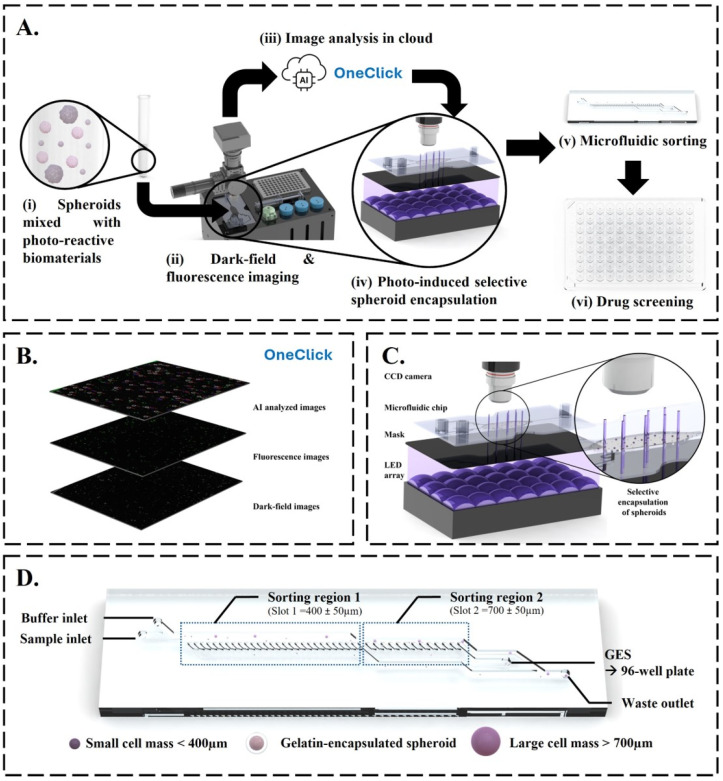
Overview of the SFSS for uniform GES generation. (**A**) Overall workflow of the GES generation process. (**B**) Cloud-based OneClick AI image analysis of different images to identify desired spheroids. (**C**) Rendering image of fabrication chamber for selective photo-crosslinking GES encapsulation. (**D**) Rendering image of microfluidic sorter chip for selective sorting of the desired cell masses.

To address the persisting challenges in producing 3D spheroids for biomedical research, we introduce the spheroid selection, fabrication, and sorting system (SFSS), an automated solution for producing uniform spheroids in a high-throughput manner (Scheme 1A). Our SFSS incorporates an advanced spheroid imaging module (Scheme 1B) and “OneClick” cloud-based AI image analysis capabilities to identify suitable spheroids (Scheme 1C) from irregular sizes and shapes of spheroids that were formed using low attachment plates. Then, through selective photo-crosslinking of biomaterial fabrication and microfluidic sorting (Scheme 1D) with precise control over size, circularity, and cell viability, uniform gelatine-encapsulated spheroids (GESs) can be generated. In this work, we demonstrate that the SFSS can streamline the spheroid production process, enhancing precision and scalability for different drug screening procedures.

## 2. Materials and Methods

### 2.1. Microfluidic Chips

#### 2.1.1. Microfabrication Chip

The three-layer microfabrication chip was an assembly of top and bottom sealing 1.5 mm thick transparent glass slides with a 0.4 mm thick polymethyl methacrylate (PMMA) spacer, whose features were laser cut by a laser engraving machine (LaserPro X500III, GCC, Taiwan, China). Two layers of 0.05 mm thick adhesive transfer tape (467MP, 3M, Minneapolis, MN, USA) and PEEK adaptors (N-1032M, Yika Technology, Shenzhen, China) were used to assemble and hold the chip under pressure. PEEK adaptors were fastened to the top cover glass. In the microfabrication chip, the biofabrication chamber (BFC) is a 250 mm^2^ window for both bioanalysis and biofabrication. Spheroids were premixed with biomaterial and injected into the BFC via the sample inlet port, and phosphate-buffered saline (PBS) entered the BFC through three 0.8 mm wide microfluidic channels with Tesla valves simultaneously to propel the biomaterial to the outlet after fabrication.

#### 2.1.2. Sorter Chip

The two-layer sorter chip enables the physical separation of the gelatine-encapsulated spheroids from cell masses, unsolidified biomaterial, and precipitates previously reported by Lu and co-workers [31]. The sorter chip was a polydimethylsiloxane (PDMS)-based polymer device replicated from a polymethyl methacrylate (PMMA) mould, constructed using CNC milling. To manufacture the sorter chip, PDMS (SYLGARD 184 silicone elastomer kit, Dow Silicones Corp., Miland, MI, USA) was added to the PMMA mould, vacuumed, and then cured in an oven at 70 °C for 4 h. After the curing, the sorter chip was bonded to a 1.5 mm single-layer glass slide by oxygen plasma treatment, ensuring a secure and leak-proof assembly. Luer adapters (IDEX L-129, Yika Technology, Shenzhen, China) were attached to the glass slide with resin (UV-sensitive resin basic, KINGROON, Shenzhen, China) to facilitate handling cell masses and collection of the GESs in the stream.

#### 2.1.3. Detection Chip

The three-layer detection chip was used to validate the labelled content in the GESs and facilitate cell mass dispensation afterward. It included 1.0 mm thick top and bottom transparent glass slides with a 0.4 mm thick PMMA spacer, whose features were laser cut by a laser engraving machine (LaserPro X500III, GCC, Taiwan). Two layers of 0.05 mm thick adhesive transfer tape (467MP, 3M, Minneapolis, MN, USA) and Luer adapters (IDEX L-129, Yika Technology, Shenzhen, China) were used to assemble the chip under pressure. Luer adapters were attached to the glass slide of the detection chip with resin (UV-sensitive resin basic, KINGROON, Shenzhen, China).

### 2.2. Manufacture of SFSS

#### 2.2.1. Imaging System and OneClick Analysis Software

The imaging system and the cloud-based OneClick analysis software V1.3 were employed for the analysis of the spheroids in the BFC in the microfabrication chip. The imaging system was designed to capture both darkfield and fluorescence images of the biomaterial within the chamber. It comprised several key components, including a charge-coupled device (CCD) camera (E3ISPM08300KPC, Tian Nuo Xiang Science Instrument, Beijing, China), a 480 nm excitation light source (LED-D1-480, Oeabt, Guangzhou, China), and a 38 mm ring light source (A3858, Ri Xin Optics, Shangrao, China). To enable fluorescence imaging, a dichroic mirror (Phtode, Beijing, China) was incorporated, along with two band pass filters (440–490 nm and 530–590 nm, Taizhu Anford Laser, Shenzhen, China) and a 400 nm long pass filter (JB400, Taizhu Anford Laser, Shenzhen, China). The optical setup was completed with an infinity plan achromatic objective lens equipped with a darkfield filter (4X, SAGA, Suzhou Shanying Optical Co., Ltd., Suzhou, China) and additional optical frameworks to ensure optimal image quality and contrast.

#### 2.2.2. The Biofabrication Module

Spheroid encapsulation process was performed in the biofabrication module of the SFSS after AI analysis of the biomaterial in the BFC. The biofabrication platform and actuation unit were the two major subsystems of the biofabrication module. The actuation unit, which facilitated the imaging process in the BFC, was composed of two linear actuators (DP150-150H, PDV, Beijing, China) and two optical limit switches (PM-L44, Sanmuron, Shenzhen, China). The biofabrication platform consisted of a syringe pump (BioArchitec Group Ltd., Hong Kong, China), a peristaltic pump (KFS-ST0B06T SI, Kamoer, Shanghai, China), an air pump (ZR320-03PM DC12V, ZhiRongHuaGuan, Dongguan, China), a PBS ceramic warmer (XH-RJ157012, XH-ELECTRON, Dongguan, China), and an optical microfabrication module (BioArchitec Group Ltd., Hong Kong, China). The syringe pump was used to transfer cell mass-embedded biomaterial to the BFC on demand.

#### 2.2.3. The Sorting System

The sorting system was composed of a spheroid sorting unit, a fluorescence detection unit, a dispenser unit, and collection tubes (50 mL glass tube, YLab, Suzhou, China) for storing the buffer and waste from the chips, and PBS. The cell mass sorting unit, consisting of peristaltic pumps (KFS-ST0B06T SI, Kamoer, Shanghai, China), a micro-pump (BioArchitec Group Ltd., Hong Kong, China), pinch valves (P20NO012-02#, BEIONFLUID, Beijing, China), and polytetrafluoroethylene (PTFE) tubes (008T32-150-10, BEIONFLUID, Beijing, China), was designed to isolate the desired GESs from the output mixture. The fluorescence detection unit, which included a photodetector (BLP-FD-101A, BLPhotons, Suzhou, China), an infinity plan achromatic objective (4X, SAGA, Suzhou, China), and a detection microfluidic chip, enabled the identification of GESs containing the desired fluorescent markers. The cell mass dispenser unit, comprising pinch valves (P20NO012-02#, BEIONFLUID, Beijing, China), PTFE tubes (008T32-150-10, BEIONFLUID, Beijing, China), an XY actuator (T6-6, PFDE, Wenzhou, China), and two optical limit switches (PM-L44, Sanmuron, Shenzhen, China), facilitated precise positioning and dispensing of individual GESs with a fixed volume of PBS into each well of a multi-well plate for further study.

### 2.3. High-Throughput Imaging of GESs by High Content Confocal Microscope

GESs were sorted into a 96-well clear flat bottom (PerkinElmer, Shelton, CT, USA) plate with a density of 1 GES per well, and the plate was incubated overnight at 37 °C in complete culture medium. Cells were imaged using the Opera Phoenix High Content Imaging System (PerkinElmer, Shelton, CT, USA). Imaging parameters of the system were set to (1) Autofocus—2 peak, (2) Optical mode—confocal, (3) Binning—2, and (4) 20× air objective lens (NA 0.4). The field of imaging was set to 69 fields per well. Stack mode was set with a vertical separation of 20 μm between planes, and the total number of planes was 8. The fluorescence channel for Calcein-AM was set to Ex 488 nm with 25% power and Em 500–550 nm with a 600 ms exposure time. The brightfield channel was set to transmission mode with 50% power and Em 650–760 nm with a 100 ms exposure time. The imaging chamber housing the culture plate was set and maintained at 37 °C in a 3% CO_2_ atmosphere spanning the entire imaging process. Images of the Calcein-AM channel were stacked to maximum projection mode and exported together with the brightfield channel using Harmony Software (Version. 4.9, PerkinElmer, Shelton, CT, USA).

### 2.4. Two-Dimensional Monolayer Cell Cytotoxicity Assay

A 100 µL volume of 1 × 10^4^ HT29 cells mL^−1^ was seeded into a 96-well plate, followed by overnight incubation at 37 °C in 5% CO_2_. After 16 h, the medium was discarded. A 10-fold serial dilution of doxorubicin (Dox) using medium was performed starting from 1 mM. Then, 100 µL of each drug was added to each well. Afterwards, the plate was placed in an incubator at 37 °C in 5% CO_2_ for 72 h. Alamar Blue viability assay was performed to evaluate the effect of drugs on the cells. All the medium was discarded from each well, and all the wells were washed twice with PBS. Then, 100 µL of 44 µM Alamar Blue solution was added to each well. The plate was incubated at 37 °C in 5% CO_2_ for 6 h. Finally, fluorescence was measured at 560/590 nm using the ThermoFisher Varioskan LUX multimode microplate reader (Thermo Fisher Scientific, Waltham, MA, USA).

### 2.5. Three-Dimensional Spheroid Cytotoxicity Assay

For spheroids produced from the SFSS, all encapsulated spheroids were automatically collected and sorted into a 96-well plate.

For spheroids produced from ultralow-attachment (ULA) plates, 100 µL of 5 × 10^4^ HT29 cells mL^−1^ was seeded into a 96-well ULA plate (Labselect, Beijing Labgic Technology Co., Ltd., Beijing, China), followed by centrifuging the plate at 1500 rcf for 3 min. Cells were allowed to develop into spheroids at 37 °C in a humidified 5% CO_2_ atmosphere. The spheroids were used directly in the 96-well ULA plate after 5 days of culturing.

For the addition of drugs, a 10-fold serial dilution of Dox using medium was performed starting from 1 mM in a 96-well plate. A 100 µL volume of each diluted drug solution was added into each well containing medium and spheroids to attain the respective concentration. The plate was then incubated at 37 °C in 5% CO_2_ for 72 h. To investigate the effect of the drugs towards the cells, an ATP bioluminescence viability assay was performed. The 3D spheroid plate and the CellTiter-Lumi Luminescent reagent (Beyotime Biotech. Inc., Shanghai, China) were equilibrated to room temperature in advance. The shaker was prewarmed to 25 °C. A 25 µL volume of CellTiter-Lumi Luminescent reagent was added into each well using a multi-channel pipette. The plate was shaken in the dark at 500 rpm for 30 min. After shaking, the solution was transferred to a white 96-well plate, and the luminescence was measured using the ThermoFisher Varioskan LUX multimode microplate reader.

### 2.6. Extraction of Spheroid Content by Enzymatic Digestion of GESs

A GES was suspended in 1 U/µL collagenase (Sigma Chemical Co., St. Louis, MO, USA) in RPMI and was incubated at 37 °C for 20 min [32]. The gel digestion was monitored in brightfield imaging using a Leica Stellaris STED confocal microscope.

### 2.7. Statistical Analysis

All values are expressed as the mean ± standard deviation from multiple technical replicates, using at least three biologically independent experiments. Student’s *t*-tests were performed for comparisons between the two groups in the FSS effect evaluation. Analysis of variance with Tukey’s honest significant difference test was performed to compare three or more groups in the toxicity assay. Differences were considered statistically significant at *p* < 0.05.

## 3. Results and Discussion

Current research in spheroid biology faces several limitations and challenges, particularly in terms of achieving high-throughput experimentation with stable and consistent results [33]. One major hurdle is the labour-intensive and time-consuming spheroid generation with methods lacking standardization in spheroid generation, leading to significant variations in spheroid size, shape, and viability. This heterogeneity can greatly impact the scalability, reproducibility, and reliability of experimental outcomes, hindering the translation of findings into clinical applications [34]. Moreover, the absence of a suitable biomaterial that can maintain the structural integrity and microenvironment of spheroids during handling and analysis further complicates the process and restricts the range of downstream applications [35]. Our innovative microfluidic platform, the SFSS, addresses these limitations by providing a fully automated, high-throughput solution for spheroid generation, encapsulation, sorting, and dispensing. The system ensures the production of highly uniform and consistent spheroids, overcoming the variability associated with conventional methods.

### 3.1. Design of the Microfabrication Chip

**Figure 1:**
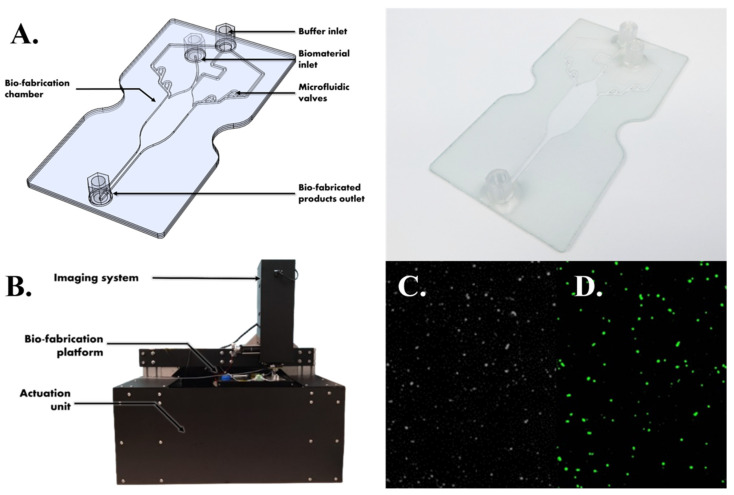
(**A**) A computer-aided design drawing and actual image of microfabrication chip. (**B**) The components of imaging unit of the SFSS. (**C**) Dark field and (**D**) fluorescence images of spheroids inside the BFC of the microfabrication chip.

The microfabrication chip is a crucial component of the microfluidic chip-based bio-fabrication system, designed to optimize the preparation, cleaning, biomaterial loading, and collection of GESs. The PMMA material was used because it offers more favourable characteristics for achieving superior optical clarity and precision in microfluidic applications. The chip architecture features two distinct inlets for buffer washing and sample introduction, with integrated Tesla valves to prevent liquid backflow. At the centre of the chip is the BFC, where the later imaging, analysis and GES fabrication processes take place (Figure 1).

### 3.2. Administration of Spheroids

Before the process, a peristaltic pump was used to introduce warm PBS from a ceramic warmer into the BFC in the microfabrication chip, facilitating thorough cleaning and washing. The PBS ceramic warmer maintained the buffer at an optimal temperature for effective chamber cleansing. Prior to biomaterial loading, an air pump was used to evacuate any residual liquid from the chamber, preventing the dilution of the biomaterial. This washing and emptying cycle was repeated twice before loading the spheroids to ensure complete removal of any unwanted debris. Subsequently, pre-mixed spheroids suspended in a photo-crosslinkable gelatine biomaterial were introduced into the BFC using a syringe pump. The microfabrication chip was securely positioned on the biofabrication platform of the SFSS (Figure 1B).

### 3.3. Imaging of Spheroids by Imaging System

After the spheroids were loaded into the BFC, images of spheroids inside the chamber were taken and followed by AI analysis. The imaging system located above the microfabrication chip was composed of a high-performance CCD camera equipped with a 480 nm excitation light-emitting diode (LED) and a set of optical filters to capture both darkfield and fluorescence images of the spheroids inside the chamber (Figure 1B). This integrated imaging system allowed for the simultaneous acquisition of darkfield and fluorescence images, providing comprehensive visual information about the biomaterial within the biofabrication chamber for subsequent analysis using the OneClick software platform. Figure 1B showcases the imaging unit of the SFSS device and its key components. The acquired imaging data clearly demonstrated the effectiveness of the system, with the darkfield image successfully capturing all debris and spheroids within the field of view (Figure 1C), while the fluorescence image selectively highlighted all viable spheroids (Figure 1D).

### 3.4. AI Image Analysis System and Spheroid Selection

**Figure 2:**
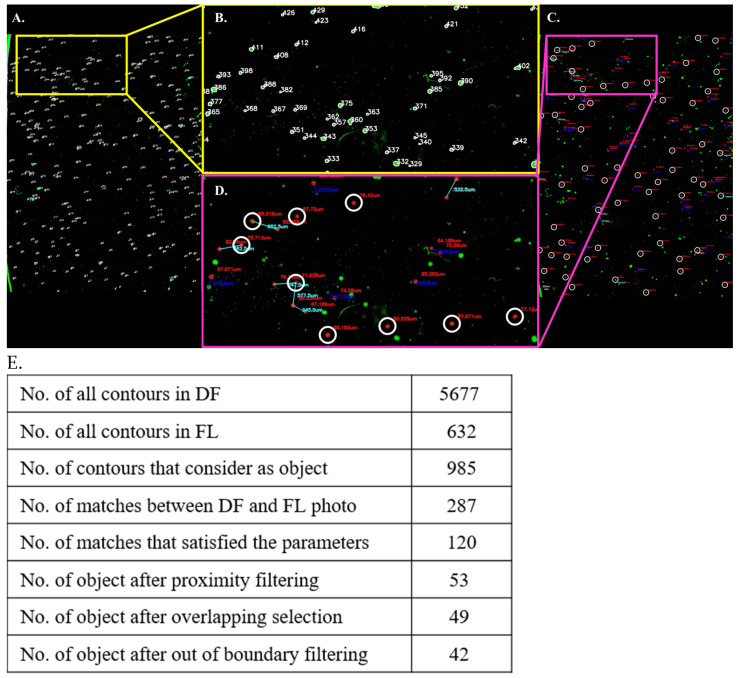
Analysis results from the OneClick program are displayed as follows: (**A**) shows the image analysis results for all objects identified within the chamber; (**B**) provides a zoomed-in view of a specific region from (**A**), with white numbers indicating the identities of objects of interest; (**C**) presents the image analysis results for spheroids selected for encapsulation; (**D**) details the extracted region from (**C**), with red text marking the radii of contours, blue lines and text showing distances between adjacent cell masses excluded due to proximity filter violations, cyan lines and text highlighting distances between overlapping cell masses excluded due to overlapping filter violations, and white circles denoting the selected cell masses that meet all criteria for encapsulation as specified in Table 1; (**E**) contains a table summarizing the total number of spheroids filtered at each step from a 200 µL sample of spheroids. DF = darkfield, FL = fluorescence.

The AI image analysis system was custom built for the spheroid selection process in the SFSS. Developed as a cloud-based solution powered by OpenCV, the open-source computer vision library called OneClick analyses and interprets captured darkfield and fluorescence images of spheroids to identify and select the most suitable candidates for encapsulation. The multi-step process begins with secure image upload, stitching, and processing in the cloud. OneClick assesses fluorescence intensity generated by the Calcein stain in the spheroid to distinguish viable spheroids and applies a series of selection filters to refine the selection process. These filters evaluate physical characteristics, spatial relationships, and coordinates within the chamber, as showed in Table 1. The analysis began with the spheroid filter to select the objects with the appropriate diameter, area, and morphology. Then, the proximity filter prevented adjacent encapsulation of multiple spheroids, maintaining adequate spacing. The overlapping filter ensured suitable separation distances between GESs, preserving their integrity and avoids unwanted bridging between two GESs. Finally, the boundary filter eliminated objects near chamber boundaries to minimize edge effect artifacts. As shown in the analysis results in Figure 2, the OneClick system generated precise coordinates for the selected spheroids through cloud computing, guiding the SFSS to prepare for the fabrication process.

### 3.5. Photo-Crosslinking and Cell Viability

The selective encapsulation of spheroids was performed through the illumination of 405 nm LED to the corresponding coordinates obtained previously inside the BFC. The light was applied at a power density of 3 mW/cm^2^ for a duration of 75 s. All selected spheroids were encapsulated simultaneously. To validate the crosslinking process, the whole microfabrication chip was viewed under a confocal microscope, and confocal images were taken. Figure S2 shows that the spheroids can be accurately encapsulated by our device, and the cells inside the spheroids remain viable. The boundary of the crosslinked biomaterial can be seen under brightfield image, where spheroids with similar diameters were located at the centre of the GESs.

### 3.6. Sorting and Dispensing of GESs

**Figure 3:**
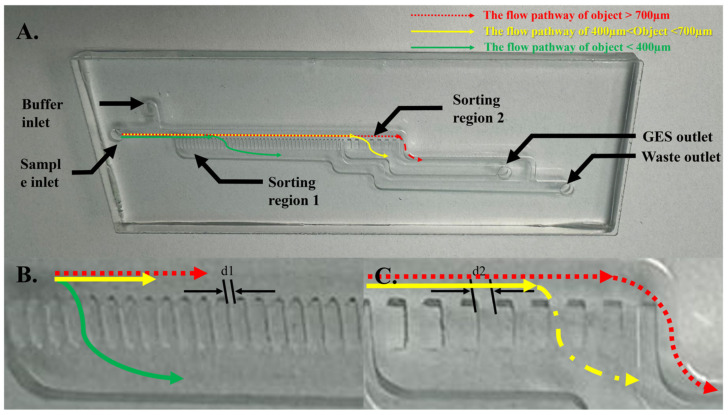
Images of the microfluidic sorter chip designed for the separation of GESs from other substances based on their size. (**A**) An image of the entire sorter chip, highlighting the inlets for the buffer and crosslinked GES stream, as well as the outlets for the sorted GESs and waste. (**B**) A magnified view of Sorting region 1, featuring slots with dimensions of d1 = 400 ± 50 µm, designed to remove objects smaller than 400 µm. (**C**) A magnified view of Sorting region 2, containing slots measuring d2 = 700 ± 50 µm, intended to collect objects ranging between 400 µm and 700 µm in size. Objects exceeding 700 µm are directed through a 1.2 mm wide microfluidic channel to the waste outlet. The red arrows show the expected route of any object with sizes larger than 700 µm, which leads to the waste outlet. The green arrows show the expected route of smaller objects with sizes of less than 400 µm, going through the slots to the waste outlet. The yellow arrows show the expected route of GESs with a size range of 400–700 µm, going through the slots to the GES outlet and entering the detection chip.

Following the selective encapsulation process, the samples were flushed out of the chamber using a pre-warmed PBS and were directed into a collection tube. The SFSS efficiently sorted and dispensed the GESs using the microfluidic chips and detection systems. The sorting chip was designed to segregate the crosslinked GESs from unwanted spheroids, cell debris, and un-crosslinked materials based on their sizes. As shown in Figure 3A, the buffer and crosslinked GES stream were introduced into the inlets of the sorter chip. The sample stream was then forced against two sorting regions composed of different sizes of slots, the design of which was previous reported in 2016 [31].

Sorting region 1 featured slots with dimensions of d1 = 400 ± 50 µm (Figure 3B), while Sorting region 2 had slots measuring d2 = 700 ± 50 µm (Figure 3C). Objects smaller than 400 µm were retained in Sorting region 1, and spheroids ranging between 400 µm and 700 µm were directed to Sorting region 2. Any substances exceeding 700 µm were channelled through a 1.2 mm wide microfluidic pathway after Sorting region 2 to the waste outlet.

**Figure 4:**
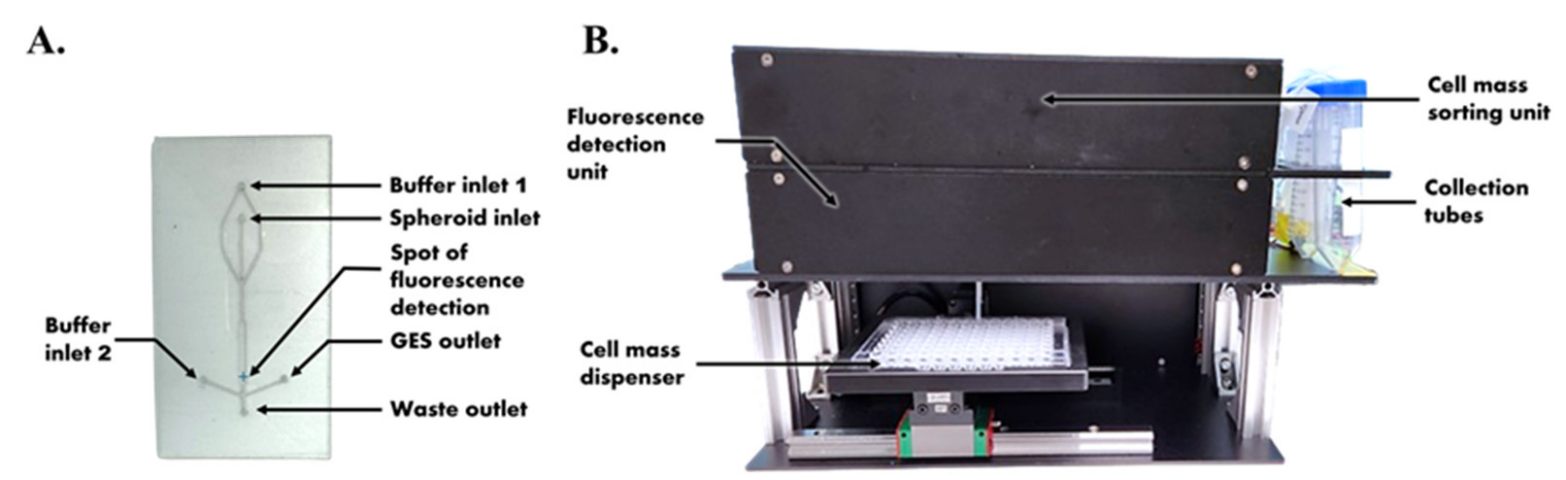
(**A**) Image of detection chip and (**B**) the dispensing unit of the SFSS. It is composed of the sorter chip, detection chip, fluorescence detector, and dispenser.

After the sorting process, the GESs reached the detection chip, where they underwent a fluorescence verification process to confirm the presence of specific markers before dispensation into the desired containers. The sorted GESs were further diluted with PBS to increase the distance between individual GESs inside the detection chip (Figure 4A). As the spheroids entered from the spheroid inlet into the microfluidic pathways, a fluorescence detection unit positioned beneath the chip continuously monitored variations in the fluorescence intensity. Figure S1 shows an example of the signal spike when a fluorescent GES passed through the detector. Upon detecting a fluorescence signal, the flow of GESs was momentarily halted, and PBS was introduced from Buffer inlet 2. This step propelled the GESs through the dispensation microchannel towards the designated GES outlet, ensuring precise and controlled handling of the biomaterial for further analysis or collection.

The outlet of the detection chip was integrated with the nozzle in the dispensation system, enabling the accurate dispensation of individual GESs into a multi-well plate. The dispensation system was equipped with a two-dimensional actuator, specifically calibrated to the dimensions of a standard 96-well plate (Figure 4B). This feature allowed for the dynamic positioning of the dispensation head over the appropriate well, ensuring the GESs were precisely and efficiently allocated to their intended locations. The integration of the sorter chip and detection chip allowed for the automated and efficient isolation, detection, and dispensing of the desired GESs from the output mixture generated by the biofabrication module, enabling seamless transfer to the multi-well plate for downstream analysis and experiments.

### 3.7. Characterisation of GESs

**Figure 5:**
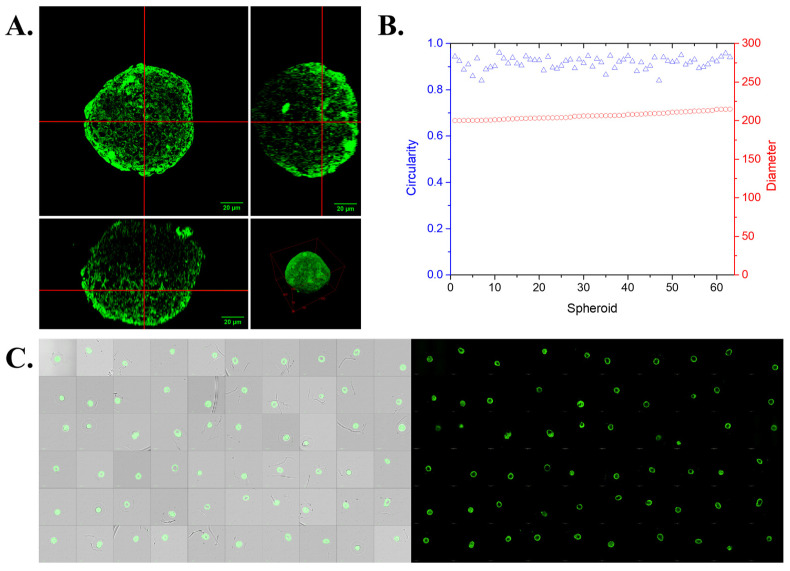
(**A**) Z-stack confocal microscopic image of an optically cleared spheroid. (**B**) The distribution of size and circularity of 66 samples of sorted spheroids. (**C**) Merged brightfield/fluorescence (**left**) and fluorescence (**right**) images of high-content whole-plate confocal tile scanning of a 96-well plate containing sorted GESs. The image was taken from a representative section of the plate, spanning from well B2 to G11, demonstrating the uniform distribution and consistent morphology of the GESs within each well.

Following the sorting process, Z-stack confocal microscopy was employed to assess the size distribution and uniformity of the sorted GESs. In Figure 5A, a 3D image of a GES demonstrates the preserved integrity and morphology of the spheroids when using the SFSS. The data revealed that the 66 sorted spheroids, each placed into individual wells, exhibited a highly uniform size distribution, ranging from 200 µm to 215 µm in diameter (Figure 5B). This narrow size range highlights the platform’s ability to produce GESs with exceptional uniformity and consistency, a crucial factor for ensuring reproducibility in downstream applications. In addition to the uniform size distribution, the sorted GESs demonstrated a high degree of circularity, with an average value of 0.9 ± 0.1. This impressive circularity score underscores the platform’s capacity to select spheroids with a near-perfect spherical shape, further emphasizing the consistency and quality of the GESs produced by the SFSS. High-content confocal microscopy was also performed on the 96-well plate to monitor the sorted GESs (Figure 5C). Our data confirmed that all the cells inside these GESs were live cells, as determined by LIVE/DEAD staining assay (Figure S3).

### 3.8. Drug Response Experiment

**Figure 6:**
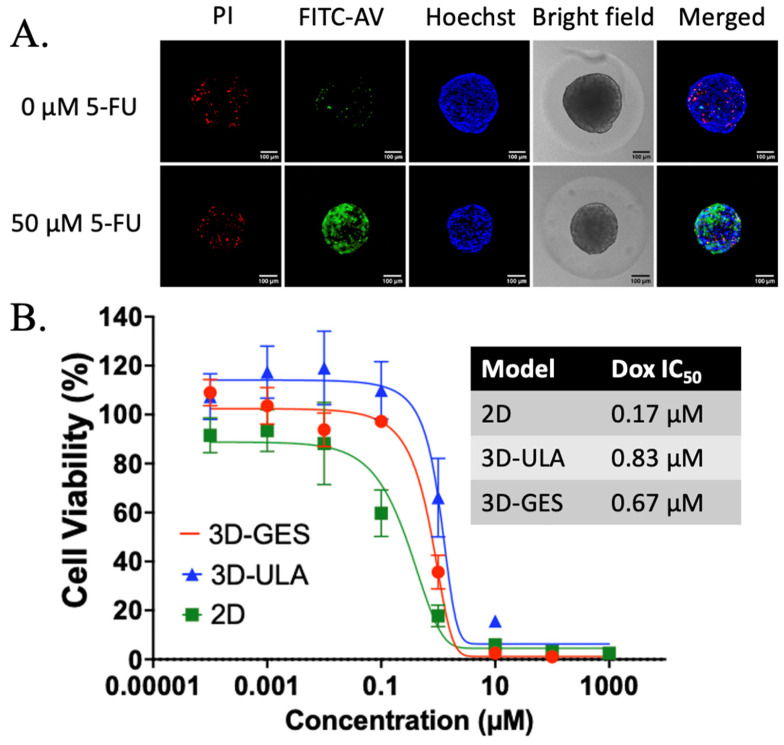
(**A**) Maximum Z-projection image of 5-FU treated and untreated HT29 GESs. (**B**) Drug–response curve of Dox treatment against monolayer 2D culture (2D), GESs (3D-GES) and 3D spheroids generated by ULA plate (3D-ULA).

To demonstrate the capability of using GESs to facilitate drug screening, we conducted drug testing experiments using 5-Fluorouracil (5-FU), a well-known apoptosis-inducing compound. The viability of the cell spheroids was assessed using annexin-V/propidium iodide staining for detecting apoptosis. Confocal microscopy analysis revealed the difference in the fluorescence signals between 5-FU-treated and untreated spheroids. The treated spheroids exhibited a strong green fluorescence signal from annexin-V, indicating the initiation of apoptosis in response to the drug treatment. In contrast, the untreated spheroids showed minimal fluorescence, confirming the specific responsiveness of the GESs to 5-FU (Figure 6A). These observations highlight the SFSS and the potential of GESs as a reliable and sensitive model for evaluating drug-induced apoptosis.

To further validate the high-throughput spheroid generation ability of the SFSS in drug screening, we constructed a dose–response curve for HT29 spheroids exposed to varying concentrations of doxorubicin (Dox). The half-maximal inhibitory concentration (IC_50_) values were determined for three different cell culture models: 2D monolayer culture, traditional 3D spheroids generated by ULA plate, and GES models. The IC_50_ for the 2D monolayer culture was found to be 0.17 µM, while the 3D spheroid models generated from ULA plate exhibited an IC_50_ of 0.83 µM, which was 5-fold higher than that of the 2D model. The GES models had an IC_50_ of 0.71 µM, demonstrating the similarity in drug response between the GES and traditional 3D spheroid models with a *p* value of 0.68, illustrating that no significant difference was observed. Notably, the ULA model exhibited higher standard deviations compared to the GES model, suggesting that the uniform size of the GESs may contribute to more reproducible and reliable drug response data (Figure 6B). The drug response experiment showcases the simplicity, speed, automation, and high-throughput capability of the SFSS in facilitating drug screening and highlights its potential to bridge the gap between 2D and 3D cell culture models. The ability of GESs to closely mimic the drug response observed in traditional 3D spheroid models, while offering the advantages of uniform size distribution and consistent microenvironment, positions them as a promising tool for high-throughput drug screening and personalized medicine applications.

### 3.9. Gelatine Digestion of GESs

Lastly, we demonstrated that our photo-crosslinked GESs could be digested to extract the spheroids from the gelatine matrix, enabling further downstream analysis and applications. The ability to regenerate spheroids from the GESs is crucial for various purposes, such as single-cell sequencing, immunostaining, and transcriptome analysis. By removing the biomaterial, we can open up more possibilities and expand the potential applications of the GES-derived spheroids. Therefore, we sought to demonstrate that our gelatine-based biomaterial could be efficiently digested by enzymes in a short time. Figure S4 illustrates the enzymatic digestion process for GESs at different time points. The time-lapse images clearly show the progressive degradation of the gelatine matrix, ultimately leading to the complete release of the encapsulated spheroids.

## 4. Conclusions

In conclusion, we have successfully designed and implemented a state-of-the-art, fully automated microfluidic SFSS. This innovative system offers a high-throughput solution for the analysis, encapsulation, sorting, and dispensing of uniform spheroids while preserving their structural integrity and morphology, thereby addressing the limitations and challenges associated with conventional manual techniques. The SFSS demonstrates a high degree of reliability and efficiency in generating large-scale, homogeneous spheroids with consistent size, shape, and viability. These characteristics make the SFSS particularly well-suited for high-throughput drug screening applications for spheroids, and even high-throughput organoid study in the future. Furthermore, the potential applications of this technology extend beyond drug screening and hold potential for a wide range of biomedical research endeavours.

## Figures and Tables

**Table 1:** Filter parameters for spheroid selections.

Parameters	Values
Min. area of the objects	78 µm^2^
Min. radius of the cells	90 µm
Max. radius of the cells	120 µm
Min. area of the cells	31,416 µm^2^
Max. area of the cells	38,013 µm^2^
Masking radius of GelMA	200 µm
Masking offset of GelMA	100 µm

## Data Availability

Data are contained within the article and Supplementary Material.

## Supplementary Materials

The following supporting information can be downloaded at: https://www.mdpi.com/article/10.3390/bios14080392/s1, Experimental Section; Figure S1. The signal generated by the detector when a fluorescent GES passed through the detector; Figure S2. Stitched confocal microscopic images of 20 representative GESs at the bio-fabrication chamber after AI selection and solidified by the 405 nm LED; Figure S3. The confocal microscopic images and LIVE/DEAD staining of GESs by our SFSS device; Figure S4. Brightfield images of GES digestion in 1 U/µL collagenase in RPMI to retrieve the spheroid from the GES.
