# Supplementary material for: Automated Uniform Spheroid Generation Platform for High Throughput Drug Screening Process
Source: Biosensors (Basel). 2024 Aug 15;14(8):392. doi: 10.3390/bios14080392 (PMC11352754; doi:10.3390/bios14080392)
Supplement: Supplementary file 1 [file biosensors-14-00392-s001.zip › biosensors-3086201-supplementary.pdf]

Supplementary Information

Automated Uniform Spheroid Generation Platform for High Throughput Drug Screening Process

Kelvin C. C. Pong [1,2], Yuen Sze Lai [3], Roy Chi Hang Wong [3], Alan Chun Kit Lee [3], Sam C. T. Chow [2],
Jonathan C. W. Lam [2], Ho Pui Ho [1,*] and Clarence T. T. Wong [3,*]

[1] Department of Biomedical Engineering, The Chinese University of Hong Kong, New Territories, Hong Kong, China
[2] BioArchitec Group Limited, Hong Kong, China
[3] State Key Laboratory of Chemical Biology and Drug Discovery, Department of Applied Biology and Chemical Technology, The Hong Kong Polytechnic University, Kowloon, Hong Kong, China

Contents

Experimental Section

Table S1 Sorting efficiency experiment materials

Table S2 Collection data from outlets

Table S3 Efficiency and specificity of the sorting chip

Figure S1. The signal generated by the detector when a fluorescent GES passed through the detector.

Figure S2. Stitched confocal microscopic images of 20 representative GESs in the biofabrication chamber after AI selection and solidified by the 405 nm LED.

Figure S3. Confocal microscopic images and LIVE/DEAD staining of GESs by our SFSS device.

Figure S4. Brightfield images of GES digestion in 1U/µL collagenase in RPMI to retrieve the spheroids from the GESs.

Cell lines and culture conditions

HT29 human colorectal adenocarcinoma cells (ATCC, no. HTB-38) were maintained in Roswell Park Memorial Institute (RPMI) 1640 medium (ThermoFisher Scientific, cat. no. 11875093) supplemented with foetal bovine serum (10%) (ThermoFisher Scientific, cat. no. A5256701) and penicillin-streptomycin (100 units mL^{-1} and 100 μg mL^{-1}, respectively, ThermoFisher Scientific, cat. no. 15140122). All the cells were grown at 37 ˚C in a humidified 5% CO_2 atmosphere.

Culture, collection and staining of spheroids grown for SFSS

HT29 cells (1.5×10^5 mL^{-1}) were resuspended in 4 mL medium, then seeded in an agarose microwell dish. The spheroids were then gently flushed and collected from the agarose microwell dish after 5 days of culturing and washed twice with PBS by centrifugation at 50 g for 1 minute. Afterwards, 1 mL of Hank's balanced salt solution (HBSS) was added and transferred to a 1.5 mL Eppendorf tube. Next, 1 μM of calcein-AM was added and incubated at 37 °C and 5% CO_2 for 15 minutes. At the same time, the number of spheroids was counted. After incubation, the spheroids were washed once using HBSS and adjusted to 20 spheroids μL-1. The spheroids were then mixed with prewarmed 20% GelMA (EFL-Tech Co. Ltd, Suzhou, China) in a 1:1 (v/v) ratio and transferred to the SFSS.

Optical clearing of spheroids

Optical clearing of the spheroids was performed using tetrahydrofuran (THF) as the dehydrating agent. The spheroids were initially immersed in a solution of 30% THF in PBS and gradually exposed to increasing concentrations of THF, reaching 100% THF over a period of 8 h. Following dehydration, the spheroids were transferred to a solution of benzyl alcohol and benzyl benzoate (1:1 v/v) and incubated overnight for refractive index matching. Z-stack confocal imaging of the optically cleared spheroids was performed using a Leica Stellaris STED confocal microscope. The resulting z-stack images were compiled and processed using ImageJ software, providing a comprehensive view of the internal structure and organization of the spheroids.

Apoptosis staining using FITC-Annexin-V and propidium iodide

HT29 spheroids were incubated in the presence and absence of 50 μM 5-FU at 37 °C in a 5% CO_2 humidified atmosphere for 72 h. The spheroids were then stained with fluorescein isothiocyanate (FITC)-Annexin V and propidium iodide (PI) using the apoptotic kit (Biosharp Life Science, Anhui, China) for 2 h and 1 μM Hoechst 33342 for 2 h at 37 °C before confocal microscopy analysis. The confocal images were taken with a Leica Stellaris STED confocal microscope and analyzed by ImageJ software.

Studies and characterisation of sorting efficiency

Experiments were conducted to determine the sorting efficiency of the sorting chip. Different sizes of polystyrene beads in phosphate-buffered saline (PBS) were sent to the sorting chip. The beads were then collected at different outlets, the sorting data were obtained, and the efficiency was calculated using the equations below.

Table S1 Sorting efficiency experiment materials

Object image	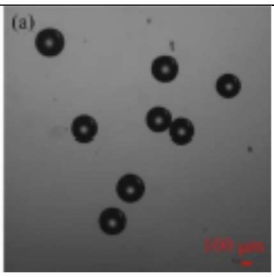	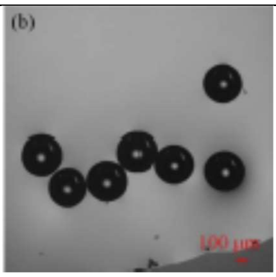	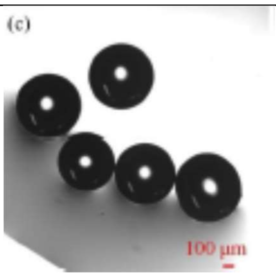
Object size	300 µm	500 µm	700 µm
Nos. of objects	30	30	30

Table S2 Collection data from outlets

Size of object collected	GES outlet	Waste outlet
300	4	26
500	29	0
700	0	26

Table S3 Efficiency and specificity of the sorting chip

Efficiency and specificity	Calculation	Result
Recovery rate, $\eta_{recovery}$	$\frac{(4+26+29+26)}{(30+30+30)} \times 100\%$	94%
Sorting efficiency, $\eta_{sorting}$	$\frac{(29)}{(30)} \times 100\%$	97%
Specificity	$\frac{(29)}{(29+4)} \times 100\%$	88%

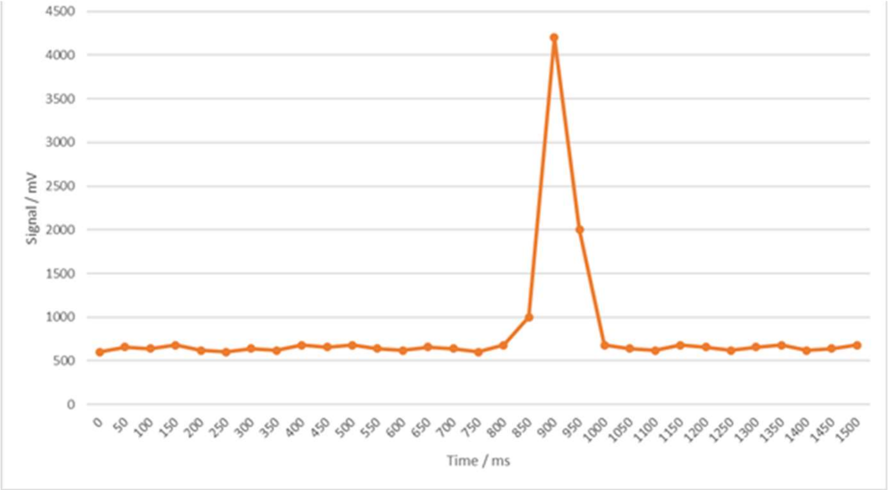

Figure S1. The signal generated by the detector when a fluorescent GES passed through the detector against time.

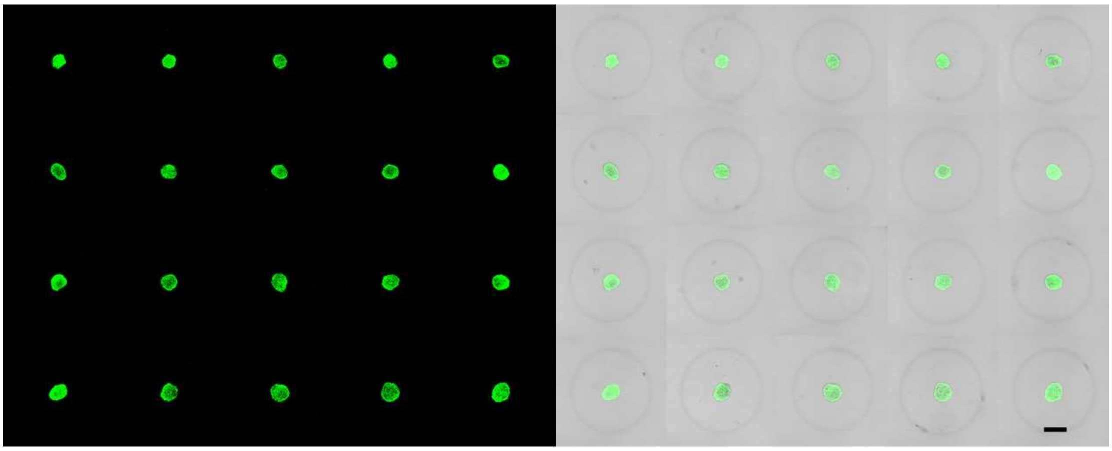

Figure S2. Stitched confocal microscopic images of 20 representative GESs in the BFC after AI selection and solidified by the 405 nm LED. The spheroids were stained with calcein-AM. Left: Green fluorescent channel. Right: Merged image of fluorescent channel and brightfield. Scale bar denotes 200 µm.

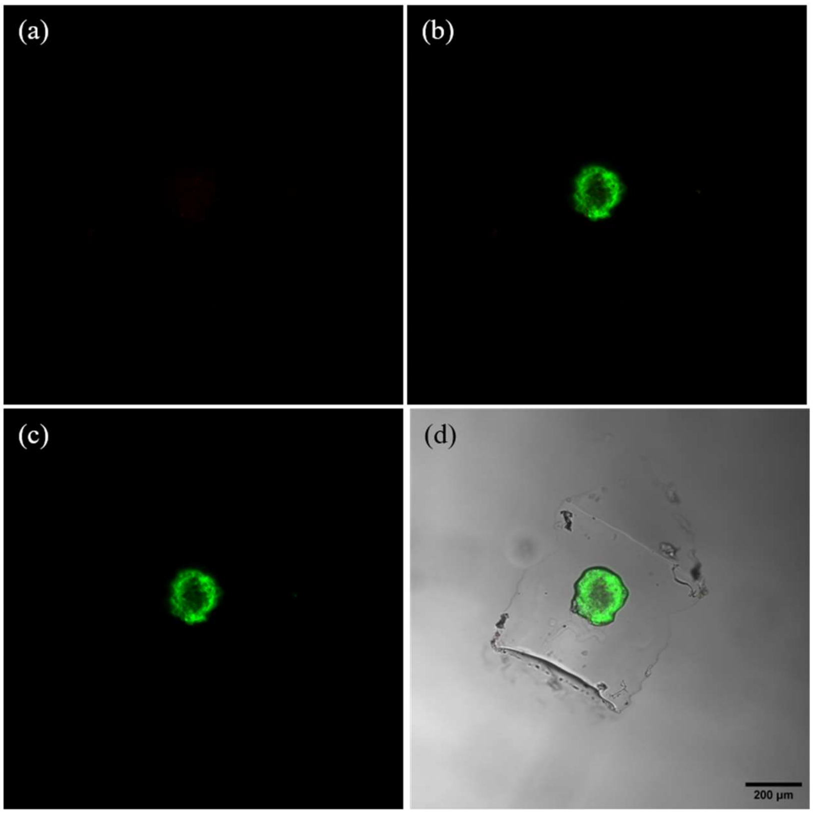

Figure S3. Confocal microscopic images and LIVE/DEAD staining of GES. Images of (a) propidium iodide staining, (b) calcein-AM staining, (c) the merged fluorescence, and (d) the combined brightfield.

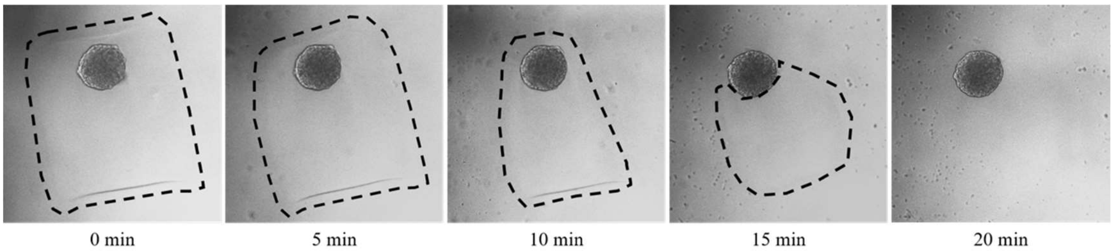

Figure S4. Brightfield images of GES digestion in 1U/μL collagenase in RPMI to retrieve the spheroids from the GESs.
